# Supplementary material for: Direct Utilization of Near-Infrared Light for Photooxidation with a Metal-Free Photocatalyst
Source: Molecules. 2022 Jun 23;27(13):4047. doi: 10.3390/molecules27134047 (PMC9268673; doi:10.3390/molecules27134047)
Supplement: Supplementary file 1 [file molecules-27-04047-s001.zip › molecules-1782670-supplementary.pdf]

Direct Utilization of Near Infrared Light for Photooxidation with Metal-Free Photocatalyst

Le Zeng, Zhonghe Wang, Tiexin Zhang * and Chunying Duan *

State Key Laboratory of Fine Chemicals, Dalian University of Technology, Dalian 116024, China

E-mail: zhangtiexin@dlut.edu.cn; cyduan@dlut.edu.cn

Table of Contents

1. Chemicals and Materials

2. Characterizations

3. Supplementary Tables and Figures

4. NMR Data and Spectra of the Products

5. References

1. Chemicals and Materials

Copper trifluoroacetate ($Cu(OTf)_2$), benzylamine, 4-methoxybenzylamine, 4-fluorobenzylamine, 2-chlorobenzylamine, 3-chlorobenzylamine, 4-chlorobenzylamine, tetrahydroisoquinoline, dibenzylamine, tris(2,2'-bipyridyl)ruthenium(II) chloride ($Ru(bpy)_3Cl_2$), dichloromethane (DCM), tetrahydrofuran (THF), methanol (MeOH), acetonitrile (MeCN), ethyl acetate (EtOAc), 1,4-diazabicyclo[2.2.2]octane (DABCO), benzoquinone (BQ), 1,3-diphenylbenzofuran (DPBF) and deuterated chloroform ($CDCl_3$) were purchased from Sigma-Aldrich. All of the above-mentioned chemicals were used as received without further purification.

2. Characterizations

^{1}H NMR spectra were measured on a Bruker 500 MHz spectrometer. UV−Vis spectra of BDP and $Ru(bpy)_3Cl_2$ were recorded on an Agilent Cary-5 spectrophotometer. Steady-state fluorescence spectra of BDP and $Ru(bpy)_3Cl_2$ were measured on a HITACHI F-7000 spectrometer. For the photooxidation, LEDs from Mightex Company were used as the excitation light source (455 nm and 720 nm) with power intensity of 20 mW/cm^2.

3. Supplementary Tables and Figures

Table S1. Optimization of reaction conditions for photooxidative benzylamine coupling.

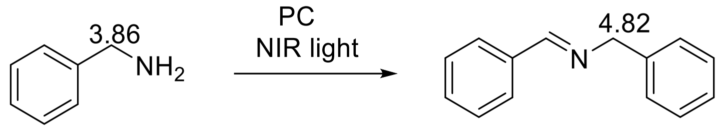

Entry	Solvent	Reaction time (h)	Conversion (%)	TON	TOF (min^{-1})
1	DCM	1	26	2.27×10^2	3.8
2	DCM	2	100	8.59×10^2	7.2
3	THF	1	32	2.71×10^2	4.5
4	THF	2	74	6.32×10^2	5.3
5	CH_3CN	2	100	8.59×10^2	7.2
6	EtOAc	2	97	8.34×10^2	7.0
7	MeOH	2	100	8.59×10^2	7.2
8[b]	DCM	2	0	0	0
9[c]	DCM	2	0	0	0
10[d]	DCM	2	0	0	0

[a] Benzylamine (0.275 mmol), photocatalyst (BDP) (1.6×10^{-4} mmol), solvent (1 mL), light (720 nm, 20 mW/cm^2), in air.
[b] Under argon atmosphere. [c] In dark. [d] No photocatalyst.

Table S2. Mechanistic study on BDP-catalyzed benzylamine oxidation under NIR irradiation.[a]

Entry	Additive	Reaction time (h)	Conversion (%)[b]	TON	TOF (min^{-1})
1[c]		0.5	31	264	8.8
2		0.5	17	143	4.8
3[d]	DABCO	2.0	0	0	0
4[e]	BQ	2.0	84	718	6.0
5		2.0	100	859	7.2

[a] Reaction condition: benzylamine (0.275 mmol), BDP (1.6×10^{-4} mmol), DCM (1 mL), 720 nm LED (20 mW/cm^2), in air, room temperature. [b] Conversion determined by ^1H NMR. [c] Solvent changed to $CDCl_3$. [d] The concentration of DABCO is 0.18 mmol/mL. [e] The concentration of BQ is 0.18 mmol/mL.

Table S3. Mechanistic study on BDP-catalyzed arylboronic acid oxidation under NIR irradiation.[a]

Entry	Additive	Reaction time (h)	Yield (%)[b]
1		4	90
2[c]		4	0
3[d]		4	0
4[e]		4	0
5[f]		4	0
6[g]	BQ	4	8

[a] Reaction condition: 4-methoxyphenylboronic acid (0.2 mmol), BDP (1.6×10^{-4} mmol), DCM/methanol (2 mL; 1/1, v/v), 720 nm LED (20 mW/cm^2), in air, room temperature. [b] Isolated yield. [c] Under argon atmosphere; [d] In dark. [e] In the absence of BDP. [f] In the absence of TEA. [g] The concentration of BQ is 0.18 mmol/mL.

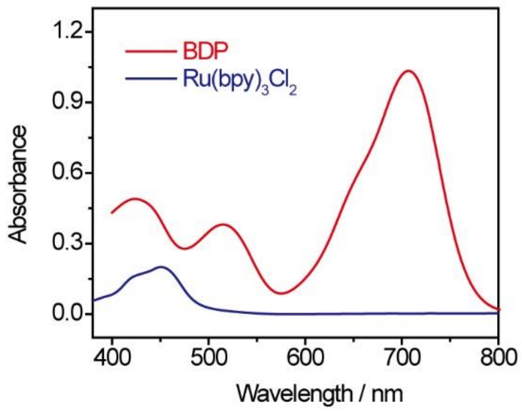

Figure S1 . UV-vis absorption spectra of $Ru(bpy)_3Cl_2$ and BDP, in DCM, 10 μM.

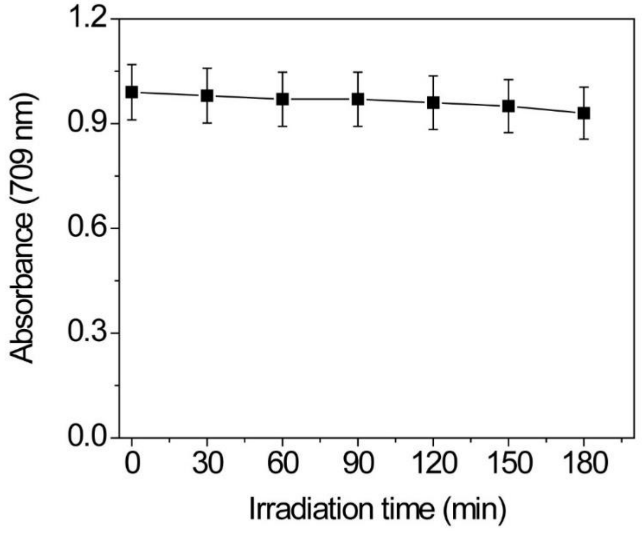

Figure S2. The absorbance change of BDP at 709 nm with 720 nm LED (20 mW/cm^2) illumination (DCM as solvent, in air).

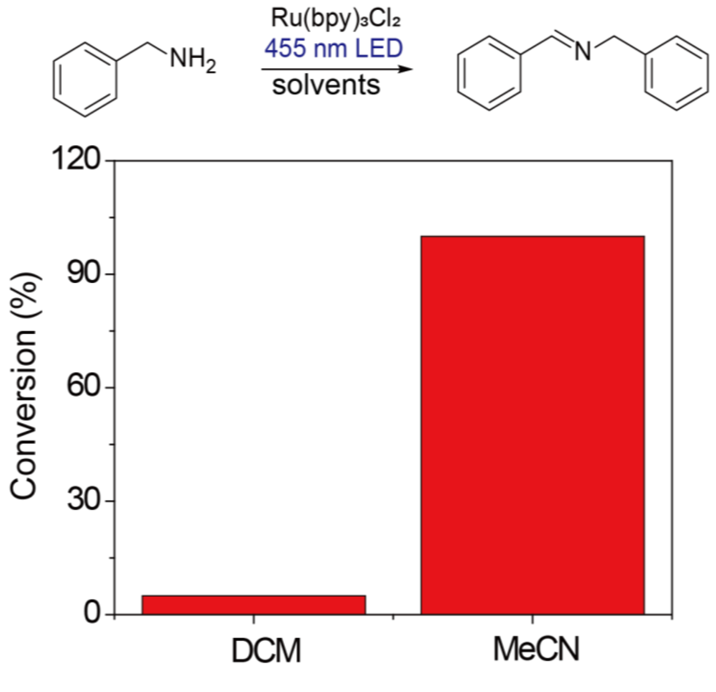

Figure S3. Visible light-irradiated benzylamine coupling by using Ru(bpy)$_3$Cl$_2$ as photocatalyst, with DCM and MeCN as the solvent, respectively. Reaction conditions: Ru(bpy)$_3$Cl$_2$ (1.6×10^{-4} mmol), benzylamine (0.275 mmol), 455 nm LED (20 mW/cm^2), solvent (1 mL).

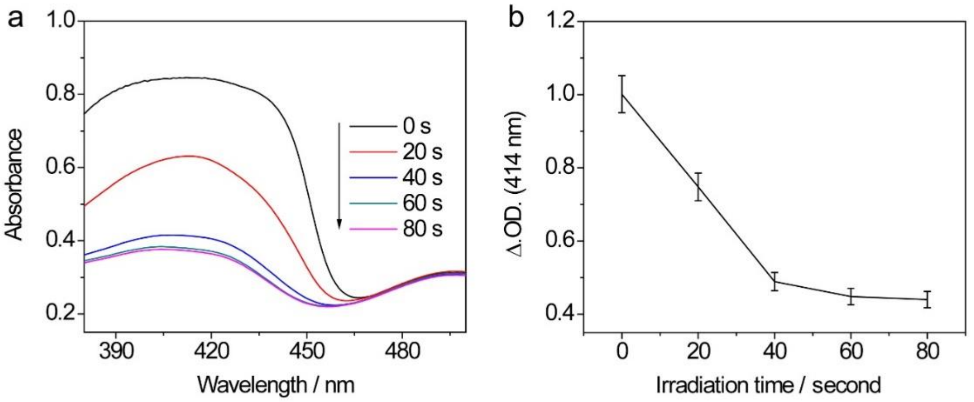

Figure S4. (a) The absorption changes of DPBF in the presence of BDP (10 μM) along with 720 nm LED irradiation. (b) The quantitative analysis of DPBF absorbance variation at 414 nm in the presence of light and BDP with DCM as the solvent.

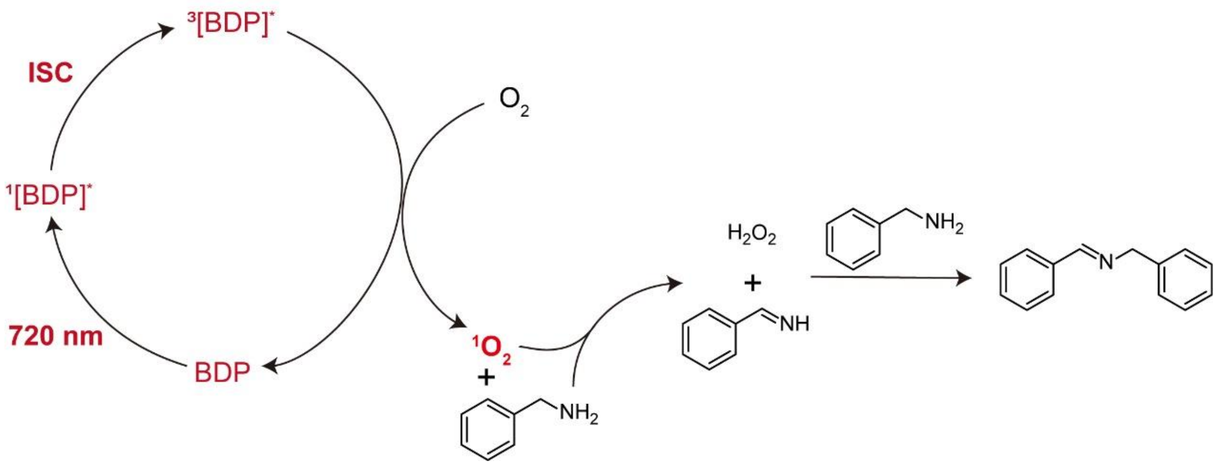

Figure S5. Proposed mechanism for the photocatalytic oxidation of benzylamine using BDP as photocatalyst under NIR irradiation.

4. NMR Data and Spectra of the Products

(*E*)-*N*-benzylidene-1-phenylmethanamine (2a) [1]

^{1}H NMR (500 MHz, $CDCl_3$) δ 8.38 (s, 1H), 7.78-7.76 (m, 2H), 7.41-7.39 (dd, *J* = 5.1, 2.0 Hz, 3H), 7.33 (d, *J* = 4.4 Hz, 4H),7.27-7.23 (m, 1H),4.81 (s, 2H).

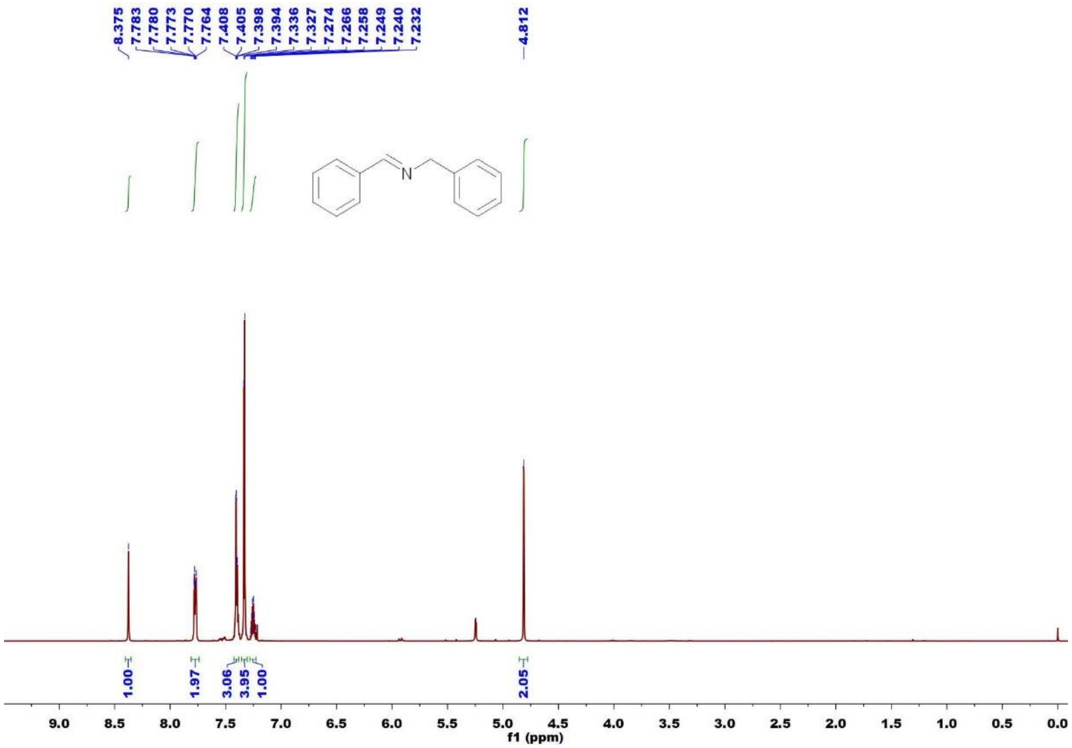

Figure S6. ^{1}H NMR spectrum of the reaction mixture of Table 1, entry 1, showing the generation of *N*-benzyl-1-phenylmethanimine (**2a**).

(*E*)-*N*-(4-methoxybenzyl)-1-(4-methoxyphenyl)methanimine (2b) [2]

^{1}H NMR (500 MHz, $CDCl_3$) δ 8.28 (s, 1H), 7.71-7.70 (d, *J* = 8.8 Hz, 2H), 7.25-7.23 (d, *J* = 8.4 Hz, 2H), 6.92-6.86 (dd, *J* = 21.0, 8.7 Hz, 4H), 4.72 (s, 2H), 3.82 (s, 3H), 3.78 (s, 3H).

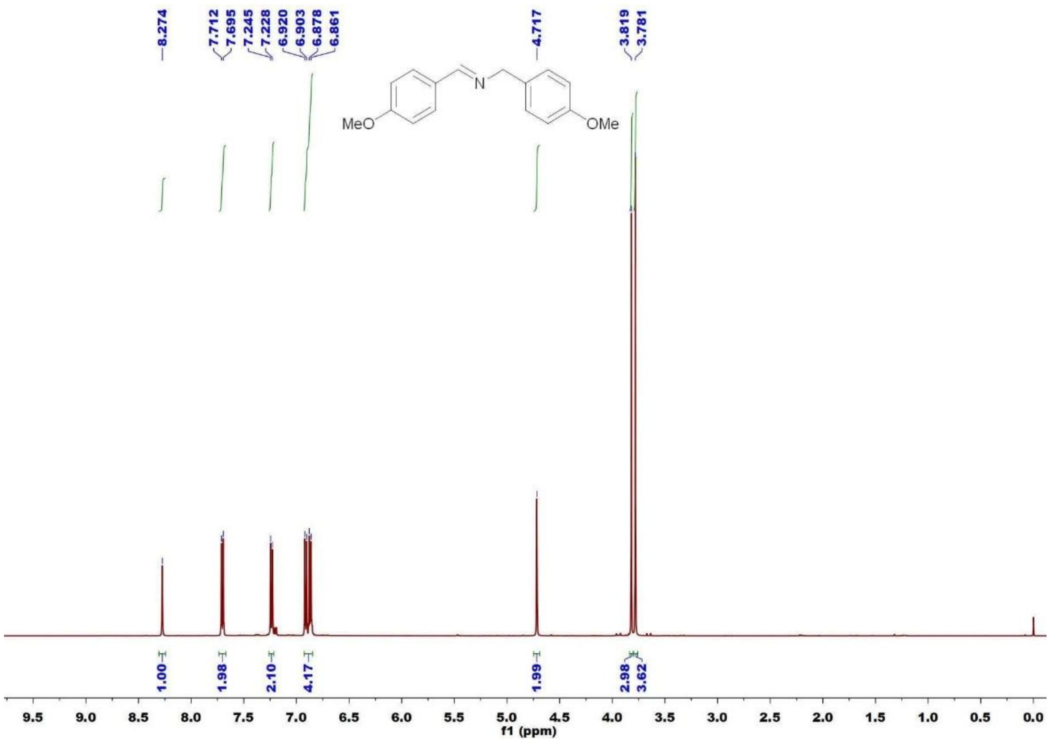

Figure S7. ^{1}H NMR spectrum of the reaction mixture of Table 1, entry 2, showing the generation of *N*-(4-methoxybenzyl)-1-(4-methoxyphenyl)methanimine (**2b**).

(*E*)-*N*-(4-chlorobenzyl)-1-(4-chlorophenyl)methanimine (2c) [2]

^{1}H NMR (500 MHz, $CDCl_3$) δ 8.32 (s, 1H), 7.71-7.69 (d, *J* = 8.4 Hz, 2H), 7.39-7.37 (d, *J* = 8.4 Hz, 2H), 7.31-7.24 (dd, *J* = 27.2, 8.4 Hz, 4H), 4.76 (s, 2H).

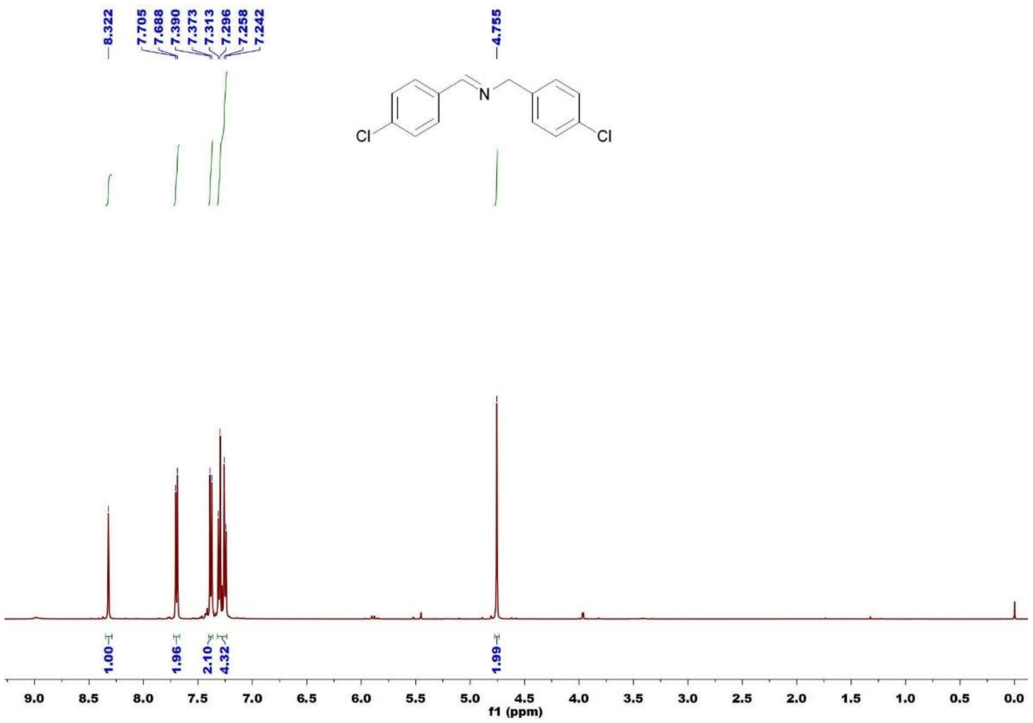

Figure S8. ^{1}H NMR spectrum of the reaction mixture of Table 1, entry 3, showing the generation of *N*-(4-chlorobenzyl)-1-(4-chlorophenyl)methanimine (**2c**).

(*E*)-*N*-(2-chlorobenzyl)-1-(2-chlorophenyl)methanimine (2d) [3]

^{1}H NMR (500 MHz, $CDCl_3$) δ 8.87 (s, 1H), 8.12-8.10 (dd, *J* = 7.7, 1.7 Hz, 1H), 7.43-7.19 (m, 7H), 4.94 (s, 2H).

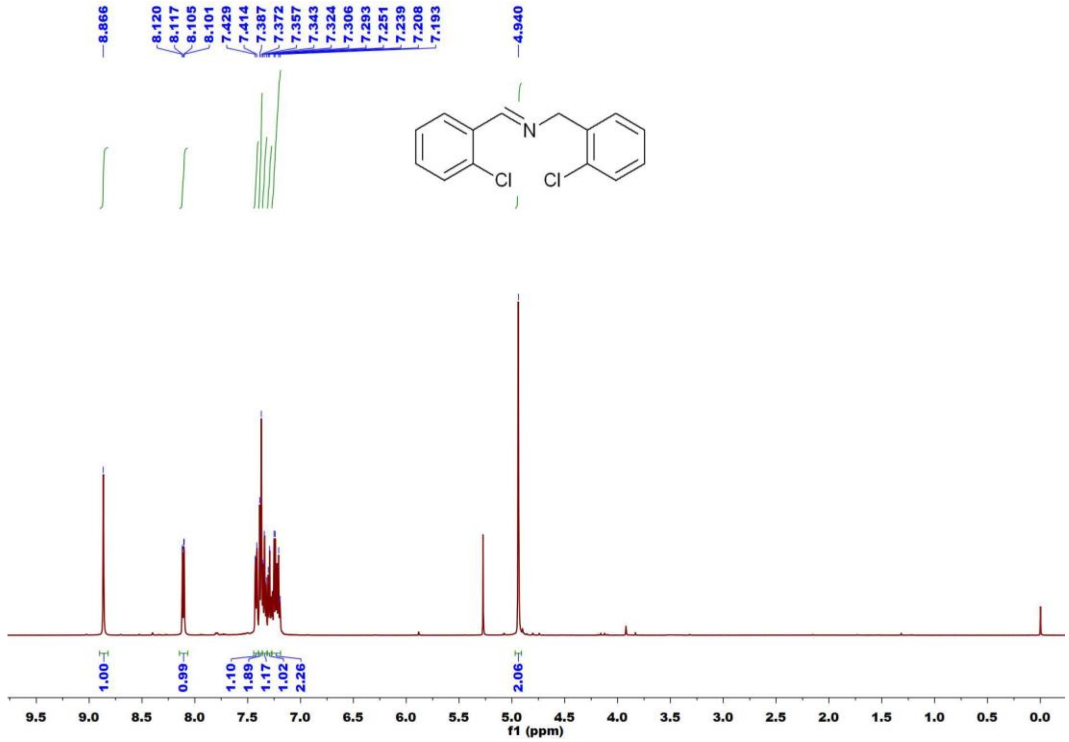

Figure S9. ^{1}H NMR spectrum of the reaction mixture of Table 1, entry 4, showing the generation of *N*-(2-chlorobenzyl)-1-(2-chlorophenyl)methanimine (**2d**).

(*E*)-*N*-(3-chlorobenzyl)-1-(3-chlorophenyl)methanimine (2e) [3]

^{1}H NMR (500 MHz, $CDCl_3$) δ 8.32 (s, 1H), 7.80 (t, *J* = 1.7 Hz, 1H), 7.62-7.61 (d, *J* = 7.6 Hz, 1H), 7.41-7.20 (m, 6H), 4.77 (s, 2H).

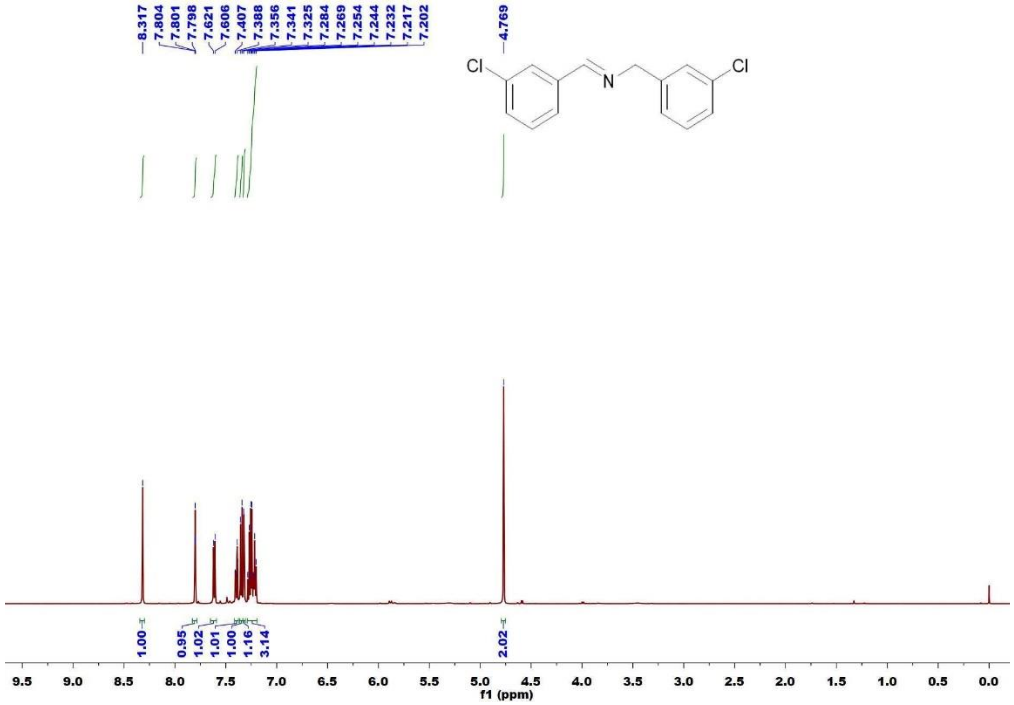

Figure S10. ^{1}H NMR spectrum of the reaction mixture of Table 1, entry 5, showing the generation of *N*-(3-chlorobenzyl)-1-(3-chlorophenyl)methanimine (**2e**).

(*E*)-*N*-(4-fluorobenzyl)-1-(4-fluorophenyl)methanimine (2f) [2]

^{1}H NMR (500 MHz, $CDCl_3$) δ 8.33 (s, 1H), 7.78-7.75 (dd, *J* = 8.8, 5.5 Hz, 2H), 7.30-7.27 (dd, *J* = 8.7, 5.5 Hz, 2H), 7.09 (t, *J* = 8.7 Hz, 2H), 7.02 (t, *J* = 8.7 Hz, 2H), 4.76 (s, 2H).

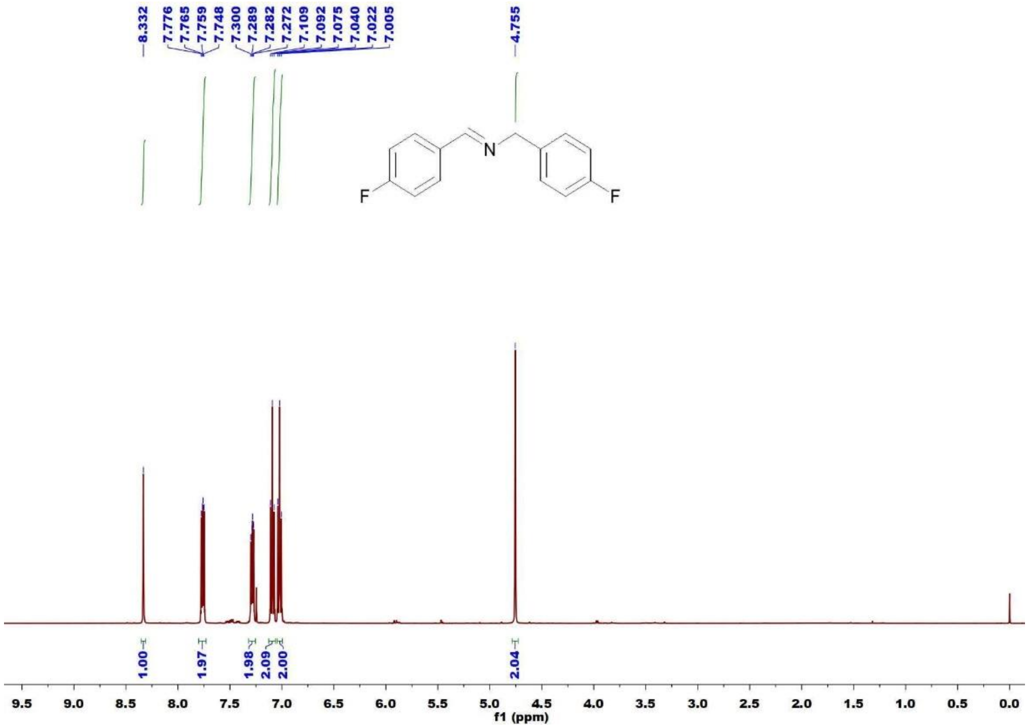

Figure S11. ^{1}H NMR spectrum of the reaction mixture of Table 1, entry 6, showing the generation of *N*-(4-fluorobenzyl)-1-(4-fluorophenyl)methanimine (**2f**).

(*E*)-*N*-(4-(trifluoromethyl)benzyl)-1-(4-(trifluoromethyl)phenyl)methanimine (2g) [4]

^{1}H NMR (500 MHz, $CDCl_3$) δ 8.46 (s, 1H), 7.91-7.89 (d, *J* = 8.1 Hz, 2H), 7.69-7.68 (d, *J* = 8.2 Hz, 2H), 7.62-7.60 (d, *J* = 8.1 Hz, 2H), 7.48-7.46 (d, *J* = 8.0 Hz, 2H), 4.89 (s, 2H).

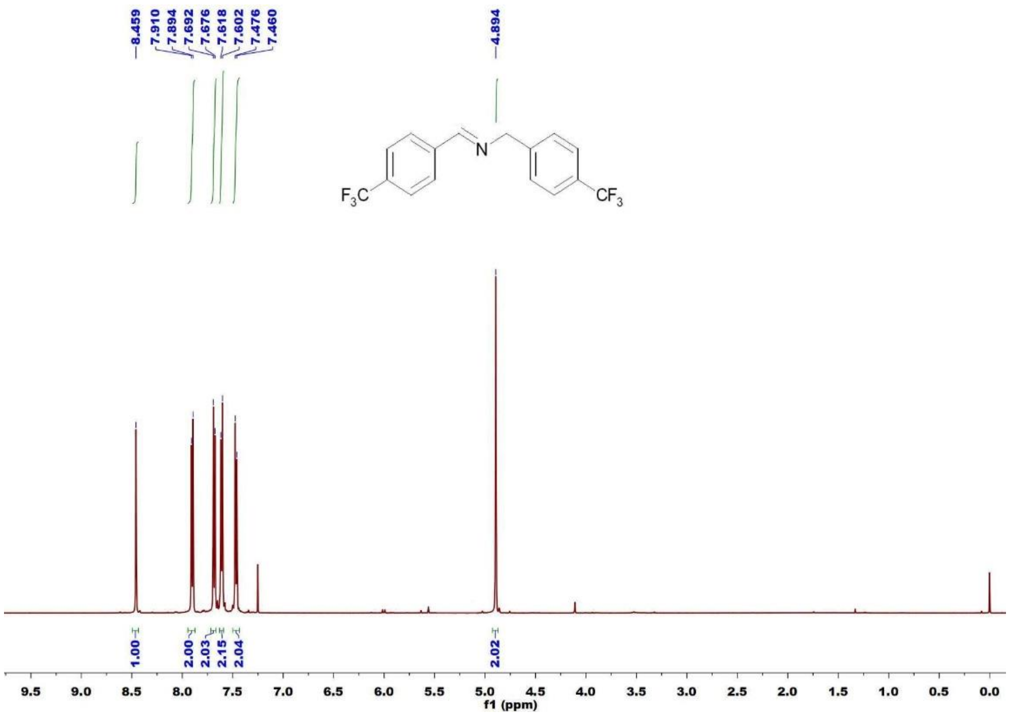

Figure S12. ^{1}H NMR spectrum of the reaction mixture of Table 1, entry 7, showing the generation of *N*-(4-(trifluoromethyl)benzyl)-1-(4-(trifluoromethyl)phenyl)methanimine (**2g**).

(*Z*)-Methyl 2-((2-methoxy-2-oxo-1-phenylethyl)imino)-2-phenylacetate (2h) [5]

^{1}H NMR (500 MHz, CD_3OD) δ 7.53 (br s, 5H), 7.45 (br s, 5H), 5.05 (s, 1H), 3.71 (s, 3H), 3.17 (s, 3H).

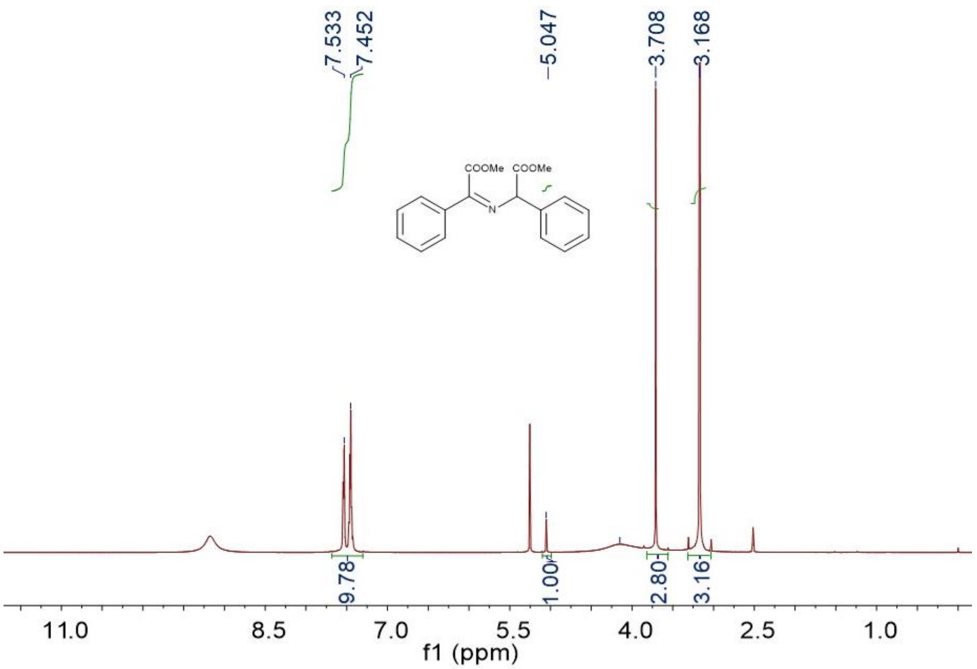

Figure S13. ^{1}H NMR spectrum of the reaction mixture of Table 1, entry 8, showing the generation of *N*-(4- methyl 2-((2-methoxy-2-oxo-1-phenylethyl)imino)-2-phenylacetate (**2h**).

3,4-Dihydroisoquinoline (2i) [6]

^{1}H NMR of product 3,4-dihydroisoquinoline (500 MHz, $CDCl_3$) δ 8.33 (s, 1H), 7.29-7.26 (t, *J* = 7.6 Hz, 2H), 7.12-7.11 (m, 2H), 3.76 (t, *J* = 6.8 Hz, 2H), 2.77-2.74 (t, *J* =7.5 Hz, 2H). The product 3,4-dihydroisoquinoline and the substrate 1,2,3,4-tetrahydroisoquinoline were not separable by flash chromatography, and the isolated yield was calculated based upon the ratio of substrate and product within ^{1}H NMR spectrum. ^{1}H NMR of substrate 1,2,3,4-tetrahydroisoquinoline (500 MHz, $CDCl_3$) δ 7.38-7.35 (t, *J* = 6.6 Hz, 1H), 7.16-7.14 (d, *J* =7.2 Hz, 1H), 7.05 (m, 1H), 6.99-6.97 (m, 1H), 4.02 (s, 2H), 3.16-3.14 (t, *J* = 6.0 Hz, 2H), 2.82-2.80 (t, *J* =6.0 Hz, 2H) [7].

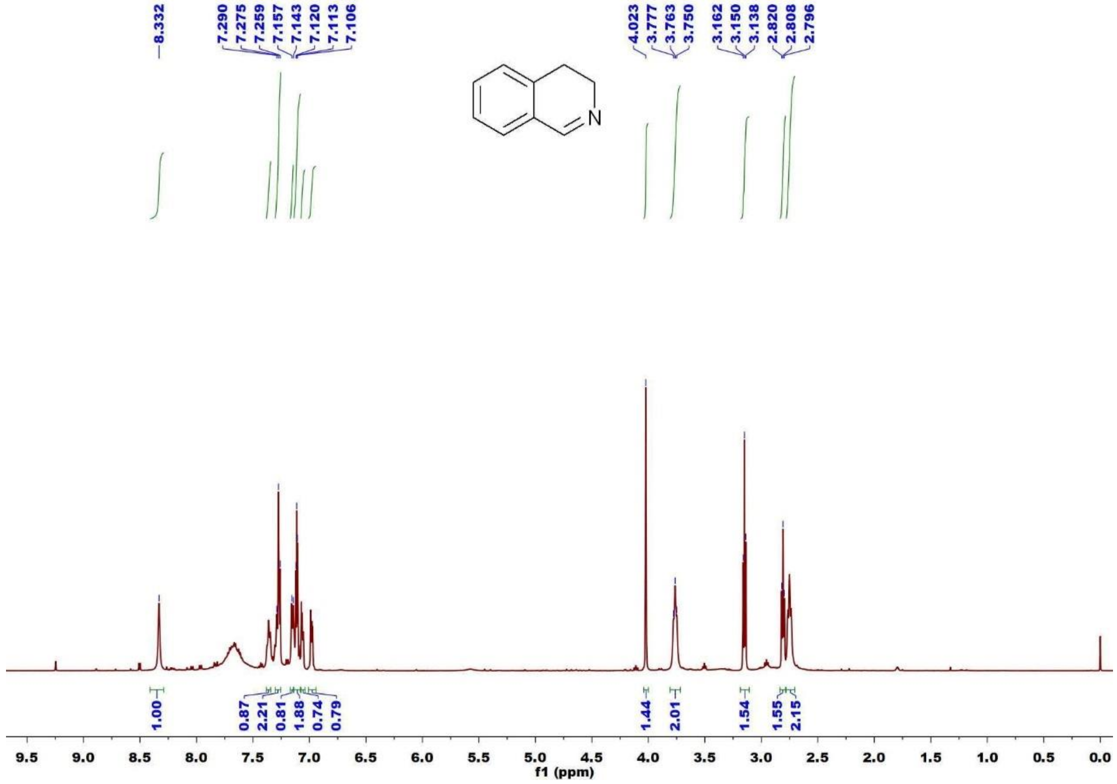

Figure S14. ^{1}H NMR spectrum of the reaction mixture of Table 1, entry 9, showing the generation of 3,4-dihydroisoquinoline (**2i**).

(*E*)-*N*-benzylidene-1-phenylmethanamine (2j) [1]

^{1}H NMR (500 MHz, $CDCl_3$) *δ* 8.38 (s, 1H), 7.78-7.76 (m, 2H), 7.41-7.40 (dd, *J* = 5.2, 2.0 Hz, 3H), 7.33 (d, *J* = 4.4 Hz, 4H),7.27-7.24 (m, 1H),4.82 (s, 2H). The NMR data were the same as compound **2a**, and also match those previously reported in the literature.

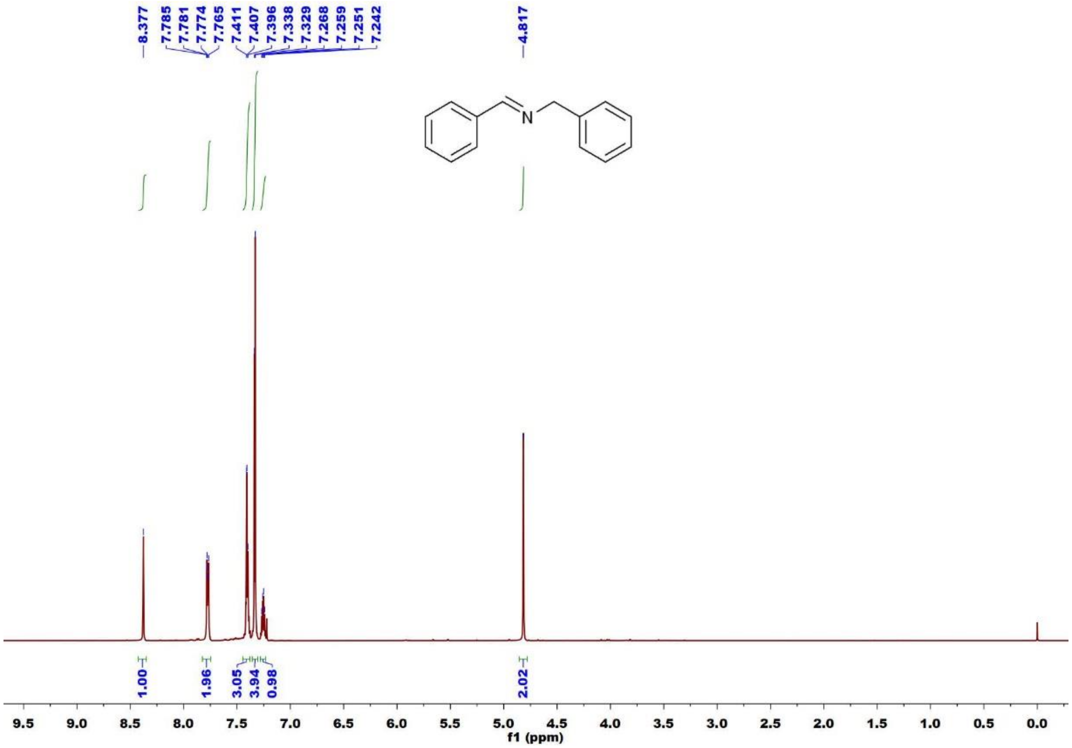

Figure S15. ^{1}H NMR spectrum of the reaction mixture of Table 1, entry 10, showing the generation of *N*-benzyl-1-phenylmethanimine (**2j**).

_N_-Benzyl-3-(4-(_tert_-butyl)phenyl)-1-phenylprop-2-yn-1-amine (10a) [8]

^{1}H NMR (500 MHz, $CDCl_3$) _δ_ 8.36 (s, 1H), 7.78-7.76 (dd, _J_ = 6.4, 2.8 Hz, 2H), 7.43-7.39 (m, 5H), 7.31 (m, 5H), 7.26-7.23 (dd, _J_ = 8.6, 4.4 Hz, 1H),4.81 (s, 2H), 3.00 (s, 1H), 1.29 (s, 9H).

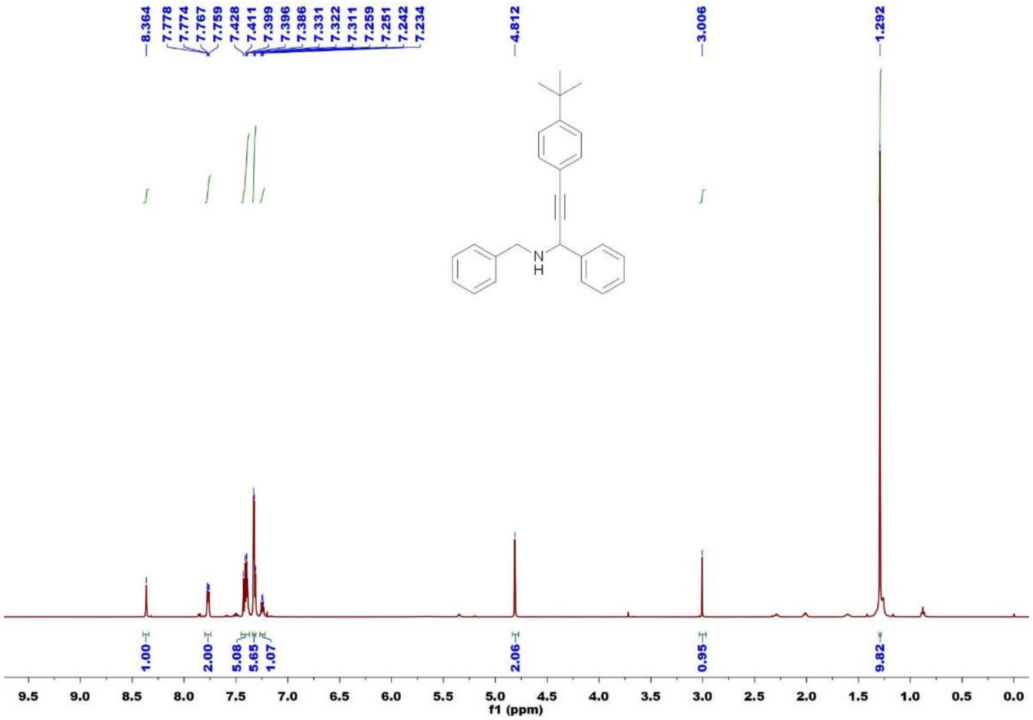

Figure S16. ^{1}H NMR spectrum of _N_-benzyl-3-(4-(_tert_-butyl)phenyl)-1-phenylprop-2-yn-1-amine, the alkyne-substituted secondary amine synthesized via the tandem catalysis of BDP and copper salt.

2-(6-Methoxynaphthalen-2-yl)propanoic acid (8a) [9]

^{1}H NMR (600 MHz, c) δ 7.69 (dd, *J* = 8.6, 2.7 Hz, 2H), 7.67 (d, *J* = 1.9 Hz, 1H), 7.40 (dd, *J* = 8.5, 1.9 Hz, 1H), 7.13 (dd, *J* = 8.9, 2.5 Hz, 1H), 7.10 (d, *J* = 2.5 Hz, 1H), 3.90 (s, 3H), 3.86 (q, *J* = 7.1 Hz, 1H), 1.58 (d, *J* = 7.2 Hz, 3H).

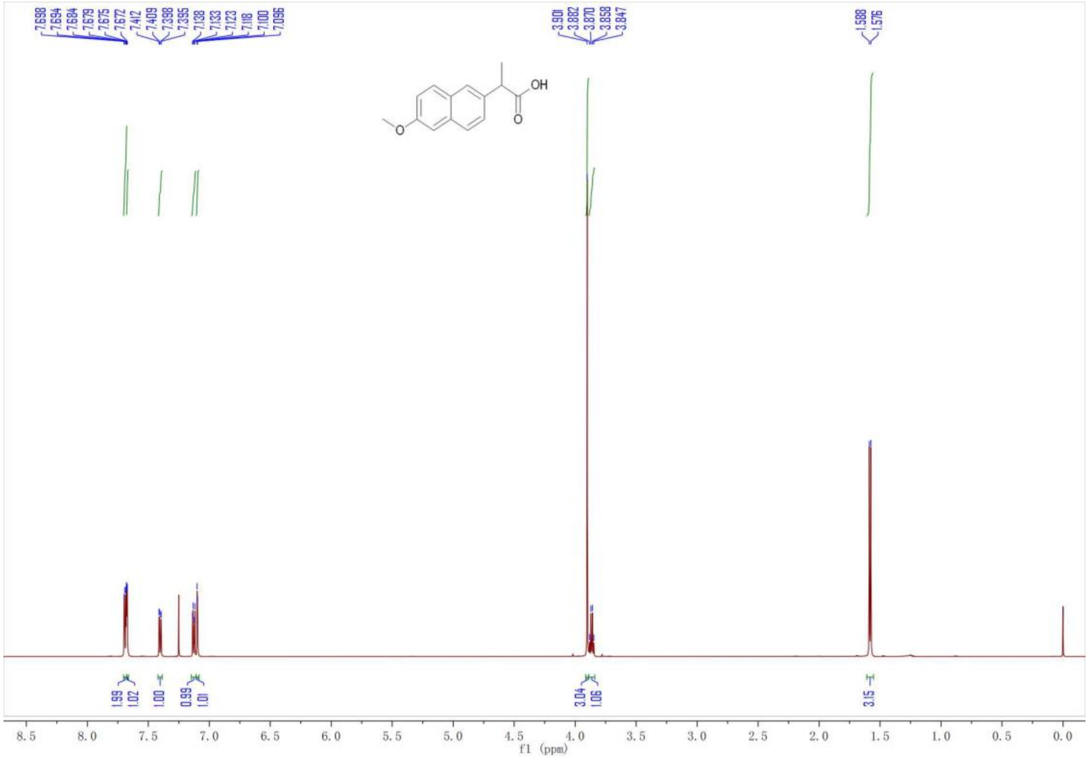

Figure S17. ^{1}H NMR spectrum of 2-(6-methoxynaphthalen-2-yl)propanoic acid, the isolated product for the BDP-catalyzed photooxidation of 4-(4,4,5,5-tetramethyl-1,3,2-dioxaborolan-2-yl)benzyl 2-(6-methoxynaphthalen-2-yl)propanoate (**7a**), in DCM/MeOH, 720 nm LED, 20 mW/cm^2, in air.

2-(1-(4-Chlorobenzoyl)-5-methoxy-2-methyl-1H-indol-3-yl)acetic acid (8b) [10]

^{1}H NMR (600 MHz, $CDCl_3$) δ 7.66-7.65 (d, *J* = 8.4 Hz, 2H), 7.47-7.46 (d, *J* = 8.4 Hz, 2H), 6.95-6.94 (d, *J* = 2.5 Hz, 1H), 6.86-6.84 (d, *J* = 9.0 Hz, 1H), 6.68-6.66 (dd, *J* = 9.0, 2.5 Hz, 1H), 3.82 (s, 3H), 3.69 (s, 2H), 2.38 (s, 3H).

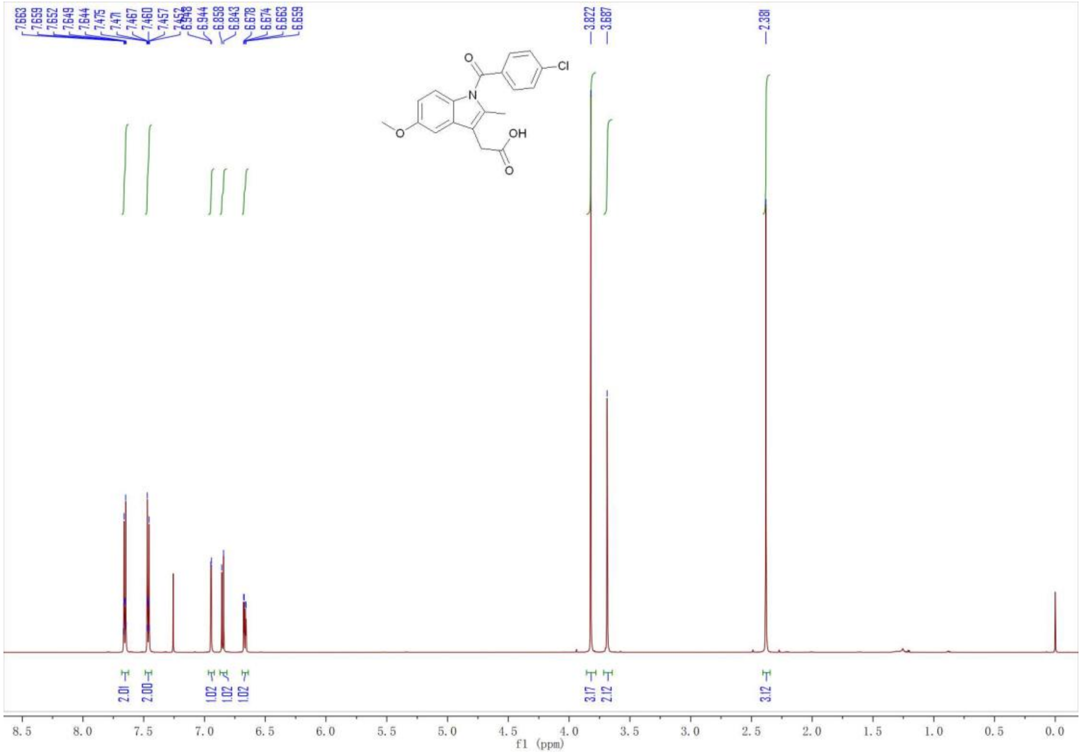

Figure S18. ^{1}H NMR spectrum of 2-(6-Methoxynaphthalen-2-yl)propanoic acid, the isolated product for the BDP-catalyzed photooxidation of 4-(4,4,5,5-tetramethyl-1,3,2-dioxaborolan-2-yl)benzyl 2-(1-(4-chlorobenzoyl)-5-methoxy-2-methyl-1*H*-indol-3-yl)acetate (**7b**), in DCM/MeOH, 720 nm LED, 20 mW/cm^2, in air.

5. References

1. Marinescu, L. G.; Pedersen, C. M.; Bols, M. Safe radical azidonation using polystyrene supported diazidoiodate(I). *Tetrahedron* **2005,** *61*, 123-127.
2. Raza, F.; Park, J. H.; Lee, H.-R.; Kim, H.-I.; Jeon, S.-J.; Kim, J.-H. Visible-Light-Driven Oxidative Coupling Reactions of Amines by Photoactive WS2 Nanosheets. *ACS Catal.* **2016,** *6*, 2754-2759.
3. Patil, R. D.; Adimurthy, S. Copper-Catalyzed Aerobic Oxidation of Amines to Imines under Neat Conditions with Low Catalyst Loading. *Adv. Synth. & Catal.* **2011,** *353*, 1695-1700.
4. Su, C.; Tandiana, R.; Tian, B.; Sengupta, A.; Tang, W.; Su, J.; Loh, K. P. Visible-Light Photocatalysis of Aerobic Oxidation Reactions Using Carbazolic Conjugated Microporous Polymers. *ACS Catal.* **2016,** *6*, 3594-3599.
5. Bradamante, S.; Colombo, S.; Pagani, G. A.; Roelens, S. The Reaction of Pyruvic Acid with Amines and Aminoesters Reexamined. Preliminary communication. *Helv. Chim. Acta* **1981,** *64*, 568-571.
6. Sasamoto, N.; Dubs, C.; Hamashima, Y.; Sodeoka, M. Pd(II)-Catalyzed Asymmetric Addition of Malonates to Dihydroisoquinolines. *J. Am. Chem. Soc.* **2006,** *128*, 14010-14011.
7. Wu, X.; Gorden, A. E. V. 2-Quinoxalinol Salen Copper Complexes for Oxidation of Aryl Methylenes. *Eur. J. Org. Chem.* **2009,** *2009*, 503-509.
8. Meyet, C. E.; Pierce, C. J.; Larsen, C. H. A Single Cu(II) Catalyst for the Three-Component Coupling of Diverse Nitrogen Sources with Aldehydes and Alkynes. *Org. Lett.* **2012,** *14*, 964-967.
9. Sharma, A.; Sharma, N.; Kumar, R.; Sharma, U. K.; Sinha, A. K. Water-promoted cascade synthesis of α-arylaldehydes from arylalkenes using N-halosuccinimides: an avenue for asymmetric oxidation using Cinchona organocatalysis. *Chem. Commun.* **2009,** 5299-5301.
10. Kasaya, Y.; Hoshi, K.; Terada, Y.; Nishida, A.; Shuto, S.; Arisawa, M. Aromatic Enamide/Ene Metathesis toward Substituted Indoles and Its Application to the Synthesis of Indomethacins. *Eur. J. Org. Chem.* **2009,** *2009*, 4606-4613.
